# The Impact of Time Use in Different Occupation Areas on Time Deficiency Among Mothers Depending on the Presence of Children With Disabilities

**DOI:** 10.1155/oti/4700309

**Published:** 2026-02-27

**Authors:** Jin-Hyuk Bang, Jong-Sik Jang, Byeong-Jin Jeon, Woo-Hyuk Jang

**Affiliations:** ^1^ Department of Occupational Therapy, College of Health Science, Kangwon National University, Samcheok, Gangwon-do, Republic of Korea, kangwon.ac.kr

**Keywords:** children with disabilities, occupation, parenting, time deficiency, time use

## Abstract

**Background:**

Previous research has documented time use patterns among mothers of children with disabilities, but few studies have examined how time allocation across specific occupational domains influences perceived time deficiency. Understanding this relationship is important for developing interventions to support these mothers′ well‐being and occupational balance.

**Purpose:**

This study is aimed at investigating differences in mothers′ time use across occupational domains based on whether their child has a disability and examining how time allocation affects perceived time deficiency.

**Methods:**

This study included 210 mothers (105 with children with disabilities and 105 with typically developing children) residing in Seoul, Gyeonggi, and Incheon, South Korea. Participants completed 24‐h time diaries, which were categorized according to the Occupational Therapy Practice Framework (fourth edition). Data were analyzed using independent samples *t*‐tests, Pearson′s correlation analysis, and multiple linear regression.

**Results:**

Mothers of children with disabilities spent significantly more time on instrumental activities of daily living (IADLs) than mothers of typically developing children (512.57 vs. 363.90 min, *p* < 0.001), particularly in child rearing (235.52 vs. 76.67 min, *p* < 0.001) and healthcare system communication (20.38 vs. 3.05 min, *p* < 0.001). Conversely, they spent less time on activities of daily living (ADLs) (142.76 vs. 165.52 min, *p* < 0.01) and work (67.05 vs. 208.10 min, *p* < 0.001). Mothers of children with disabilities reported significantly higher levels of time deficiency (3.09 ± 0.67 vs. 2.61 ± 0.75, *p* < 0.001). Among mothers of children with disabilities, increased time spent on ADLs significantly reduced time deficiency (*B* = −0.004, *p* < 0.001), whereas IADL time showed a positive but nonsignificant association with time deficiency.

**Conclusion:**

Mothers of children with disabilities experience occupational imbalance characterized by substantially increased caregiving demands and reduced personal care time, leading to heightened time deficiency. These findings highlight the need for caregiver support services and occupational balance interventions to improve these mothers′ quality of life and well‐being.

## 1. Introduction

The birth of a child brings great joy and happiness to parents but also brings new roles and responsibilities [1]. However, in modern societies, married women′s participation in the labor force has increased; consequently, their working hours have increased, and the burden of childcare and household activities has not decreased [2]. With the birth of a child, mothers have to juggle many roles simultaneously, including childcare, household work, and paid work, making it difficult for them to balance their time [3]. In addition, mothers experience various difficulties such as increased physical fatigue due to childcare, increased expenditures, and lack of personal time [4].

When a child is born with a disability, the challenges for parents are compounded [5]. Parenting a child with a disability is more challenging than parenting a child without a disability because children with disabilities have various health issues, and as they grow up, they often have behavioral, emotional, and social problems [6]. Moreover, parenting a child with a disability requires more effort than parenting a child without a disability, as their treatment, rehabilitation, and participation in special education require more time and money [7]. Because their children′s disabilities require constant care, parents find it difficult to combine work and parenting, resulting in financial and time difficulties [8].

Time is a finite resource that everyone is given equally, with only 24 h in a day [9]. Managing daily time effectively across multiple life domains is important for maintaining health and wellness [10]. The fourth edition of the Occupational Therapy Practice Framework (OTPF) defines “occupation” as personalized and meaningful participation in activities of daily living (ADLs) for a given individual. The framework categorizes the domains of occupation into the following: ADLs, instrumental activities of daily living (IADLs), health management, rest and sleep, education, work, play, leisure, and social participation [11]. In occupational therapy, occupational balance refers to how individuals perceive the distribution and mix of activities in their daily routines [12]. A well‐balanced occupational life includes appropriate proportions of required versus chosen activities, tasks that energize versus those that deplete, and pursuits oriented toward self‐care versus caring for others [13, 14]. Research shows strong connections between balanced occupational engagement and positive outcomes in health, quality of life, and psychological well‐being [15, 16]. Mothers raising children with disabilities face challenges in maintaining this balance, largely because intensive caregiving responsibilities dominate their daily schedules [10, 17]. Much of these mothers′ days involve IADLs, with child‐related tasks far exceeding those of typical parenting [18]. For these mothers, IADL tasks include scheduling and attending medical visits, overseeing therapeutic programs, navigating educational systems and advocating for appropriate services, and providing specialized daily care tailored to their child′s needs [19, 20]. This concentration of time and energy on caregiving often results in insufficient attention to the mothers′ own ADLs—basic personal care activities such as nutrition, hygiene, and rest—which are important for preventing caregiver stress and exhaustion [19].

Recent research has shown that mothers of children with disabilities have significantly poorer occupational balance compared to mothers of typically developing children [10, 15]. Studies show that these mothers experience notable disparities: an imbalance between mandatory and discretionary activities, inadequate time for personal needs, and reduced engagement in recreational and social activities [10, 21]. A study of Chinese mothers in Australia, Singapore, and Taiwan found that mothers caring for children with disabilities face higher caregiving demands, which compromise their participation in health‐promoting activities and negatively affect their overall well‐being [20]. Evidence also shows that when mothers limit their involvement in health‐promoting behaviors—including regular physical activity, adequate sleep, and self‐care routines—they report higher psychological distress and stress levels [19].

Studies examining factors that shape caregiving time distribution have shown that the child′s functional capabilities, the mother′s work status, and family economic circumstances significantly influence how mothers allocate time between caregiving and other life domains [18]. However, while existing research has documented that mothers of children with disabilities spend more time on caregiving and less on personal activities, few studies have systematically examined how time distribution across distinct occupational categories—as defined in the OTPF—affects mothers′ perceptions of time adequacy.

Time deficiency is a psychological condition in which an individual feels that they have less time than they need [22]. The amount of time that can be appropriately allocated and used in a finite amount of time varies depending on the individual′s circumstances, and the less time an individual has under his or her control, the more he or she will feel inadequate in using it [23]. Feelings of time deficiency negatively impact physical and mental health and life satisfaction [24]. Mothers with children are more likely to feel time‐poor because they have multiple roles and perform household chores in addition to raising their children [3]. In addition, the importance of time as a resource is gaining attention in modern society; consequently, the number of people who feel time‐poor is increasing [25].

Therefore, the present study is aimed at categorizing occupation areas based on the latest OTPF (fourth edition) to identify the difference in time use among mothers with and without children with disabilities and determine the effect of time use on the subjects′ feelings of time deficiency. By analyzing associations between objective time allocation across occupational categories and subjective time deficiency, this study is aimed at identifying specific areas that could be targeted through interventions to improve occupational balance and reduce caregiver stress. In this study, we sought to present the imbalanced distribution of occupation among mothers of children with disabilities and provide a foundation for understanding how it affects their perception of time deficiency. The following hypothesis was formulated for the purpose of this study.

Firstly, we predict that there will be differences in mothers′ time use across occupation areas based on whether their child has a disability or not.

Secondly, it is predicted that mothers′ feelings of time deficiency will be amplified when their child has a disability.

Thirdly, we hypothesize that there will be differences in mothers′ time deficiency based on their general characteristics.

## 2. Methods

### 2.1. Study Subjects

This study was conducted using a questionnaire survey of mothers of children with disabilities and mothers of children without disabilities living in Seoul, Gyeonggi, and Incheon. These three regions (Seoul, Gyeonggi, and Incheon) collectively form the Seoul Capital Area, South Korea′s most densely populated metropolitan region, home to approximately half of the country′s population and offering the most comprehensive healthcare and special education services for children with disabilities. The sample size was calculated using the G‐Power 3.1 program. The minimum sample size was 210 subjects, with 105 mothers per group, at an effect size of 0.5, a significance level of 0.05, and a 95% power level. Parents aged 19 years or older with minor children who voluntarily agreed to participate in the study were included in the survey, whereas those who had difficulty reading or writing in Korean or were unwilling to participate in the study were excluded. The characteristics of the study subjects were categorized by age, education level, number of children, and economic activity. This study was approved by the Institutional Review Board of Kangwon National University (KWNUIRB‐2023‐07‐005‐001).

### 2.2. Study Tool

#### 2.2.1. Time Diary

The study used a modified version of the time diary from Statistics Korea′s Living Time Survey, a nationally representative survey conducted every 5 years to understand how people allocate their 24‐h day. The time diary divides a 24‐h period into 10‐min intervals, allowing participants to record activities that lasted more than 5 min using a retrospective approach. Participants were asked to complete the time diary for one typical weekday, recording all activities from midnight to midnight. The Korean version of the time diary was used for all participants.

#### 2.2.2. Occupation Area

The study classified the behaviors of the subjects according to the OTPF (fourth edition). The occupation domains classified are ADLs, IADLs, health management, rest and sleep, education, work, leisure, and social participation. ADLs are routine and regular activities that involve caring for one′s body and include eating, applying makeup, and showering. IADLs are activities that support daily living at home and in the community, such as child rearing, cleaning the house, caring for pets, and getting around. Healthcare includes behaviors that take care of one′s own health, such as seeing a doctor and exercising to improve physical fitness. The education was divided into two categories: formal and informal. Formal education was defined as school attendance, while all other educational activities were classified as informal education. Leisure included a variety of leisure activities such as playing games, watching videos, and knitting. The behavior classification by domain is presented in Table 1.

**Table 1:** Occupation classification.

Occupations	Activities
Activities of daily living	Eating
	Personal maintenance

Instrumental activities of daily living	Child rearing
	Home management
	Care of pets and animals
	Locomotion

Health management	Social and emotional health promotion and maintenance
	Communication with the healthcare system
	Physical activity

Rest and sleep	Rest
	Sleep

Education	Formal educational
	Informal educational

Work	Job performance and maintenance
	Volunteer

Leisure	Leisure participation

Social participation	Community participation
	Family participation

#### 2.2.3. Time Deficiency

Time deficiency is defined as a psychological state in which individuals feel that they have less time than they actually need [22]. It can also be expressed as time pressure and time stress [26]. Time deficiency has been defined in the literature as “the perception that people do not have enough time to do all the things they want and need to do” [27] and in another study as “the feeling that time as a resource is insufficient compared to the desire and need for activities to be performed” [28]. To summarize these definitions, we can say that even if the absolute amount of time is small, the lack of time spent on the behaviors we want to do leads to feelings of time deficiency. This is because people prioritize how they divide their time so that they can spend more time on activities they consider important [28]. In this study, we used the Korean version of the Time Deficiency Scale from Statistics Korea′s Living Time Survey. This is a single‐item measure using a 4‐point Likert scale instrument, ranging from 1 (*I always feel like I have enough time*) to 4 (*I always feel like I don′t have enough time*), with higher scores indicating greater time deficiency. As a single‐item measure, internal consistency reliability is not applicable. However, this item has been widely used in national surveys and has demonstrated validity in previous research examining time use patterns in Korean populations.

### 2.3. Study Procedure

The study was conducted with the consent and approval of the children′s institution for data collection. The study period was from November 2023 to May 2024, and the survey was administered by an occupational therapist. A total of 227 subjects were surveyed online and in person, and after excluding 17 questionnaires with incomplete or missing time logs, 210 questionnaires were used for the final analysis. To safeguard the anonymity of subjects, we meticulously excluded any information that could potentially identify individuals. In the context of the online survey, confidentiality was preserved by implementing access restrictions, thereby ensuring that only the researcher had the ability to view the survey data.

### 2.4. Statistical Analysis

The data collected in this study were analyzed using SPSS Windows Version 25.0. The general characteristics and time usage of the study subjects were presented using frequency analysis and descriptive statistics. The temporal allocation of mothers is expected to vary according to their children′s disability status, with higher time demands placed on mothers with disabled children. To this end, an independent sample *t*‐test was conducted to compare the differences in time usage and mothers′ time deficiency by having or not having a child with a disability. The temporal demands placed upon mothers are known to vary according to a number of general characteristics. Pearson′s correlation analysis was conducted to ascertain the relationship between time usage and general characteristics by occupation area and time deficiency. Multiple linear regression analysis was conducted to determine whether time usage and general characteristics by occupation area affect time deficiency.

## 3. Results

### 3.1. General Characteristics of the Subjects

The study covered 210 subjects, with 105 mothers in each group, classified based on whether their children had a disability or not. Fifty‐eight of the children have cerebral palsy (55.2%), 39 have a developmental disability (37.2%), and eight have a genetic syndrome (7.6%). The majority of the mothers (109, 51.9%) are aged 40. There are 111 mothers with two children (52.9%), 87 (41.4%) with one child, and 12 (5.7%) with three or more children. Most respondents (110) are from Seoul (52.4%), and there are 67 from Gyeonggi (31.9%) and 33 from Incheon (15.7%). Most respondents are university graduates (89, 42.4%), while only 18 had a master′s degree or higher (8.5%). In terms of economic activity, 97 (46.2%) were employed. Table 2 details the study subjects′ general characteristics.

**Table 2:** General characteristics.

Characteristics		Disability (*n* = 105)	Normal (*n* = 105)	Total (*n* = 210)
Disability	CP	58 (55.2)	0 (0)	58 (27.6)
	DD	39 (37.2)	0 (0)	39 (18.6)
	Syndrome	8 (7.6)	0 (0)	8 (3.8)
	Normal developmental	0 (0)	105 (100.0)	105 (50.0)

Age	20~29	1 (1.0)	5 (4.8)	6 (2.9)
	30~39	59 (56.2)	28 (26.7)	87 (41.4)
	40~49	44 (41.9)	65 (61.9)	109 (51.9)
	50~59	1 (1.0)	7 (6.7)	8 (3.8)

Child	1 person	46 (43.8)	41 (39.0)	87 (41.4)
	2 persons	54 (51.4)	57 (54.3)	111 (52.9)
	3 or more persons	6 (4.8)	7 (6.7)	12 (5.7)

Area	Seoul	60 (57.1)	50 (47.6)	110 (52.4)
	Gyeonggi	30 (28.6)	37 (35.2)	67 (31.9)
	Incheon	15 (14.3)	18 (17.1)	33 (15.7)

Education level	High school	21 (20.0)	26 (24.7)	47 (22.4)
	College	34 (32.4)	22 (21.0)	56 (26.7)
	University	41 (39.0)	48 (45.7)	89 (42.4)
	Master′s degree or higher	9 (8.6)	9 (8.6)	18 (8.5)

Job status	Yes	33 (31.4)	64 (61.0)	97 (46.2)
	No	72 (68.6)	41 (39.0)	113 (53.8)

Abbreviations: CP, cerebral palsy; DD, developmental disabilities.

### 3.2. Comparing Mothers′ Time Use by Occupation Area by Whether Their Child Has a Disability or Not

After comparing mothers′ time use by occupation domain according to the presence or absence of a child with a disability, we found that mothers spend 148.67 min more time on IADLs (*p* < 0.001), 22.76 min less time on ADLs (*p* < 0.01), and 141.05 min less time on work (*p* < 0.001) when their child has a disability (Table 3).

**Table 3:** Time usage of mothers by occupation area according to the child′s disability (unit: Minute).

Area	Disability (*n* = 105)	Normal (*n* = 105)	*t*
	*M* *e* *a* *n* ± *S* *D*	*M* *e* *a* *n* ± *S* *D*	
Activities of daily living	142.76 ± 60.77	165.52 ± 54.63	−2.854 ^∗∗^
Eating	89.62 ± 44.24	103.43 ± 40.14	−2.369 ^∗^
Personal maintenance	53.14 ± 29.59	62.10 ± 33.87	−2.040 ^∗^
Instrumental activities of daily living	512.57 ± 140.03	363.90 ± 125.66	8.097 ^∗∗∗^
Child rearing	235.52 ± 110.96	76.67 ± 71.11	12.351 ^∗∗∗^
Home management	189.14 ± 86.27	189.05 ± 111.58	0.007
Care of pets and animals	2.29 ± 14.43	2.00 ± 9.65	0.169
Locomotion	96.10 ± 58.52	96.19 ± 57.89	−0.012
Health management	87.14 ± 80.37	72.10 ± 87.28	1.300
Social and emotional health promotion and maintenance	0.29 ± 1.67	3.81 ± 18.26	−1.969
Communication with the healthcare system	20.38 ± 48.20	3.05 ± 14.01	3.539 ^∗∗∗^
Physical activity	66.48 ± 77.51	65.24 ± 83.41	0.111
Rest and sleep	483.33 ± 90.60	487.81 ± 92.13	−0.355
Rest	22.57 ± 41.19	18.48 ± 33.53	0.790
Sleep	460.76 ± 84.33	469.33 ± 87.83	−0.721
Education	1.14 ± 7.11	4.95 ± 35.74	−1.071
Formal education	0.00 ± 0.00	0.00 ± 0.00	
Informal education	1.14 ± 7.11	4.95 ± 35.74	−1.071
Work	67.05 ± 130.94	208.10 ± 214.88	−5.744 ^∗∗∗^
Job performance and maintenance	65.05 ± 131.51	206.76 ± 215.95	−5.743 ^∗∗∗^
Volunteer	2.00 ± 8.59	1.33 ± 9.71	0.527
Leisure	87.62 ± 79.86	92.86 ± 73.90	−0.493
Leisure participation	87.62 ± 79.86	92.86 ± 73.90	−0.493
Social participation	50.48 ± 52.25	44.76 ± 60.77	0.731
Community participation	49.05 ± 51.69	43.14 ± 61.05	0.756
Family participation	2.86 ± 12.99	1.62 ± 10.20	0.768

Abbreviation: SD, standard deviation.

^∗^ *p* < 0.05,  ^∗∗^ *p* < 0.01, and  ^∗∗∗^ *p* < 0.001.

In terms of subscales, mothers with a child with a disability spent 158.85 min more on child rearing (*p* < 0.001) and 17.33 min more on communication with the healthcare system (*p* < 0.001) than mothers without a child with a disability. In contrast, mothers without a child with a disability spent 13.81 min more on eating (*p* < 0.05), 8.96 min more on personal maintenance (*p* < 0.05), and 141.71 min more on job performance and maintenance (*p* < 0.001) (Table 3).

### 3.3. Differences in Mothers′ Time Deficiencies Between Children With and Without Disabilities

A significant difference in perceptions of time deficiency by whether their child had a disability or not was noted, with a time pressure score of 3.09 when their child had a disability and 2.61 when their child did not have a disability. Hence, mothers felt more time‐poor when their child had a disability than when their child did not (Table 4).

**Table 4:** Difference in mothers′ perception of time deficiency based on the presence of the child′s disability.

	Disability (*n* = 105)	Normal (*n* = 105)	*t*
	*M* *e* *a* *n* ± *S* *D*	*M* *e* *a* *n* ± *S* *D*	
Time deficiency	3.09 ± 0.67	2.61 ± 0.75	−4.851 ^∗∗∗^

Abbreviation: SD, standard deviation.

^∗^ *p* < 0.05,  ^∗∗^ *p* < 0.01, and  ^∗∗∗^ *p* < 0.001.

### 3.4. Correlates of Time Use and Time Deficiency by Mothers′ Occupation Area by Child′s Disability

#### 3.4.1. Correlates of Time Use and Time Deficiency by Occupation Area in Mothers of Children With Disabilities

When examining the correlation between time use and time deficiency among mothers of children with disabilities by occupation area, time deficiency and IADLs had the highest positive correlation with *r* = 0.305 and *p* = 0.001, indicating a moderate correlation [29]. On the contrary, time deficiency and ADLs had the highest negative correlation with *r* = −0.443 and *p* ≤ 0.001 (Table 5). This indicates a moderate correlation [29].

**Table 5:** Correlation between time use by occupation area and perception of time deficiency among mothers of children with disabilities.

	Time deficiency	ADL	IADL	Health management	Rest and sleep	Education	Work	Leisure	Social participation
Time deficiency	1								
ADL	−0.443 ^∗∗∗^	1							
IADL	0.305 ^∗∗^	−0.257 ^∗∗^	1						
Health management	−0.003	−0.184 ^∗^	−0.190 ^∗^	1					
Rest and sleep	−0.002	−0.039	−0.279 ^∗∗^	−0.091	1				
Education	0.040	−0.027	0.046	0.006	−0.015	1			
Work	−0.204 ^∗^	0.105	−0.206 ^∗^	−0.136	−0.313 ^∗∗^	0.069	1		
Leisure	0.058	−0.094	0.029	0.097	−0.294 ^∗∗^	−0.117	−0.110	1	
Social participation	0.134	−0.165 ^∗^	−0.053	−0.145	0.247 ^∗∗^	−0.095	−0.230 ^∗∗^	0.013	1

Abbreviations: ADLs, activities of daily living; IADLs, instrumental activities of daily living.

^∗^ *p* < 0.05,  ^∗∗^ *p* < 0.01, and  ^∗∗∗^ *p* < 0.001.

#### 3.4.2. Correlates of Time Use and Time Deficiency by Occupation Area Among Mothers of Children Without Disabilities

When we examined the correlation between time use and time deficiency by occupation domain among mothers of children without disabilities, we found that time deficiency and education had the highest positive correlation, with *r* = 0.073, but the difference was not significant. On the other hand, time deficiency and rest and sleep had the highest inverse correlation among the correlations, with *r* = −0.186, which did not show any significant difference (Table 6).

**Table 6:** Correlation between time use by occupation area and perception of time deficiency among mothers of nondisabled children.

	Time deficiency	ADL	IADL	Health management	Rest and sleep	Education	Work	Leisure	Social participation
Time deficiency	1								
ADL	0.039	1							
IADL	0.028	−0.065	1						
Health management	−0.142	−0.252 ^∗∗^	0.112	1					
Rest and sleep	−0.186	−0.079	0.060	0.142	1				
Education	0.073	−0.080	0.055	0.009	−0.166	1			
Work	0.062	−0.038	−0.711 ^∗∗∗^	−0.439 ^∗∗∗^	−0.376 ^∗∗∗^	−0.135	1		
Leisure	−0.141	−0.027	0.114	−0.001	−0.138	0.075	−0.409 ^∗∗∗^	1	
Social participation	−0.041	−0.072	0.081	−0.109	−0.178	−0.003	−0.254 ^∗∗^	0.183	1

Abbreviations: ADLs, activities of daily living; IADLs, instrumental activities of daily living.

^∗^ *p* < 0.05,  ^∗∗^ *p* < 0.01, and  ^∗∗∗^ *p* < 0.001.

### 3.5. Effects of Mothers′ Time Use by Occupation Area on Feelings of Time Deficiency According to Whether Their Children Have a Disability or Not

#### 3.5.1. Effects of Time Use by Occupation Area on Time Deficiency Among Mothers of Children With Disabilities

A multiple linear regression analysis was conducted to determine whether the amount of time spent in each occupation area influences time deficiency among mothers of children with disabilities. The method of analysis was entered. The results of the analysis presented that the regression model was appropriate with *F* = 4.160 (*p* < 0.001), and daily activities had a significant negative effect on time deficiency with *B* = −0.004 (*p* < 0.001). Thus, it can be concluded that if the time use of daily activities increases by 1 min, the time deficiency decreases by 0.004 (Table 7).

**Table 7:** Impact of time use by occupation area on perception of time deficiency among mothers of children with disabilities.

	Unstandardized coefficients		Standardized coefficients	*t*(*p*)	TOL	VIF
	*B*	SE	*β*			
Constant	3.505	0.732		4.786		
ADL	−0.004	0.001	−0.392	−3.962 ^∗∗∗^	0.792	1.262
IADL	0.001	0.000	0.154	1.391	0.629	1.590
Health management	−0.001	0.001	−0.061	−0.603	0.761	1.314
Rest and sleep	0.000	0.001	−0.035	−0.311	0.601	1.663
Education	0.003	0.008	0.036	0.407	0.971	1.029
Work	−0.001	0.001	−0.142	−1.313	0.663	1.508
Leisure	0.000	0.001	0.000	0.004	0.824	1.213
Social participation	0.001	0.001	0.049	0.505	0.832	1.202
*F*(*p*)	4.160 ^∗∗∗^					
Adj.*R* ^2^	0.257					
Durbin–Watson	2.071					

Abbreviations: ADLs, activities of daily living; IADLs, instrumental activities of daily living.

^∗^ *p* < 0.05,  ^∗∗^ *p* < 0.01, and  ^∗∗∗^ *p* < 0.001.

#### 3.5.2. Effects of Time Use by Occupation Area on Feelings of Time Deficiency Among Mothers of Children Without Disabilities

A multiple linear regression analysis was conducted to determine whether time use by occupation area influences time deficiency among mothers of children without disabilities. The method of analysis was entered. The results of the analysis showed no significant effect of time usage by work area on time deficiency in this regression model with *F* = 1.158 (*p* > 0.05) (Table 8).

**Table 8:** Impact of time use by occupation area on perception of time deficiency among mothers of nondisabled children.

	Unstandardized coefficients		Standardized coefficients	*t*(*p*)	TOL	VIF
	*B*	SE	*β*			
Constant	3.212	0.553		5.811		
ADL	0.000	0.001	−0.008	−0.081	0.914	1.094
IADL	0.000	0.001	0.046	0.461	0.960	1.042
Health management	−0.001	0.001	−0.139	−1.348	0.900	1.111
Rest and sleep	−0.001	0.001	−0.166	−1.618	0.904	1.106
Education	0.001	0.002	0.033	0.329	0.956	1.047
Leisure	0.001	0.001	0.131	1.303	0.940	1.064
Social participation	−0.001	0.001	−0.114	−1.116	0.915	1.093
*F*(*p*)	1.158					
Adj.*R* ^2^	0.077					
Durbin–Watson	2.329					

Abbreviations: ADLs, activities of daily living; IADLs, instrumental activities of daily living.

^∗^ *p* < 0.05,  ^∗∗^ *p* < 0.01, and  ^∗∗∗^ *p* < 0.001.

### 3.6. Effects of Mothers′ General Characteristics on Feelings of Time Deficiency According to Whether Their Children Have a Disability or Not

#### 3.6.1. Effects of General Characteristics on Time Deficiency Among Mothers of Children With Disabilities

A multiple linear regression analysis was conducted to determine whether general characteristics of mothers of children with disabilities influence time deficiency. The method of analysis was entered. As a result of the analysis, *F* = 1.515 (*p* > 0.05), there was no significant effect of the general characteristics of the subjects on time deficiency in this regression model. Increasing mothers′ age had a positive effect on time deficiency, and the number of children had a negative effect on time deficiency. Differences in place of residence, education, and economic activity had no effect on time deficiency (Table 9).

**Table 9:** Impact of general characteristics on perception of time deficiency among mothers of children with disabilities.

	Unstandardized coefficients		Standardized coefficients	*t*(*p*)	TOL	VIF
	*B*	SE	*β*			
Constant	2.059	0.597		3.448		
Age	0.030	0.013	0.235	2.340	0.920	1.087
Child	−0.196	0.115	−0.170	−1.703	0.925	1.082
Seoul	0.139	0.202	0.104	0.688	0.408	2.454
Gyeonggi	0.076	0.214	0.052	0.354	0.434	2.304
High school	0.057	0.266	0.034	0.214	0.360	2.779
College	0.192	0.252	0.136	0.763	0.293	3.415
University	0.289	0.245	0.213	1.181	0.285	3.503
Job status	−0.288	0.159	−0.202	−1.808	0.744	1.344
*F*(*p*)	1.515					
Adj.*R* ^2^	0.112					
Durbin–Watson	1.939					

*Note:* Reference group: area∗Incheon, education level∗master′s degree or higher, and job status∗no.

^∗^ *p* < 0.05,  ^∗∗^ *p* < 0.01, and  ^∗∗∗^ *p* < 0.001.

#### 3.6.2. Effects of General Characteristics on Feelings of Time Deficiency Among Mothers of Children Without Disabilities

A multiple linear regression analysis was conducted to determine whether general characteristics of mothers of children with disabilities influence time deficiency. The method of analysis was entered. As a result of the analysis, *F* = 1.296 (*p* > 0.05), there was no significant effect of the general characteristics of the subjects on time deficiency in this regression model. Increasing mothers′ age had a positive effect on time deficiency, and the number of children had a negative effect on time deficiency. Differences in place of residence, education, and economic activity had no effect on time deficiency (Table 10).

**Table 10:** Impact of general characteristics on perception of time deficiency among mothers of nondisabled children.

	Unstandardized coefficients		Standardized coefficients	*t*(*p*)	TOL	VIF
	*B*	SE	*β*			
Constant	2.348	0.601		3.908		
Age	0.008	0.013	0.069	0.658	0.863	1.159
Child	−0.054	0.128	−0.043	−0.425	0.915	1.093
Seoul	0.053	0.212	0.036	0.251	0.469	2.132
Gyeonggi	0.162	0.221	0.103	0.733	0.474	2.111
High school	−0.177	0.300	−0.102	−0.591	0.316	3.164
College	−0.481	0.298	−0.261	−1.614	0.359	2.788
University	−0.288	0.275	−0.191	−1.046	0.282	3.552
Job status	0.339	0.157	0.220	2.157	0.901	1.110
*F*(*p*)	1.296					
Adj.R^2^	0.097					
Durbin–Watson	2.425					

*Note:* Reference group: area∗Incheon, education level∗master′s degree or higher, and job status∗no.

^∗^ *p* < 0.05,  ^∗∗^ *p* < 0.01, and  ^∗∗∗^ *p* < 0.001.

## 4. Discussion

This study is aimed at investigating mothers′ time use according to the presence or absence of their children′s disabilities and to examine how time allocation across occupational domains affects their time deficiency. To this end, we examined time use by modifying the time diary of the Living Time Survey conducted by Statistics Korea and compared the subjects′ occupational engagement by categorizing occupation areas according to the OTPF fourth edition. The main findings of this study are as follows.

First, we examined mothers′ time use by occupation area according to whether their child had a disability or not and found that mothers with a child with a disability spent 22.76 min less on ADLs than mothers without a child with a disability, with 13.81 min less on eating and 8.96 min less on personal maintenance. These findings align with previous studies showing that children with disabilities are more likely to have eating problems than children without disabilities and that parents of children with disabilities are constrained in their mealtimes because of assisting their children to eat [30]. In addition, studies have revealed that parents spend a lot of time caring for their children with disabilities, not only during mealtime but also during their personal time, which inevitably reduces their physiological time [31]. Time is a finite resource, and the more time spent on one activity, the less time they have for other activities [32]. Mothers of children with disabilities may have spent more time in other areas of their lives and therefore have less time for their own personal ADLs. However, a decrease in the amount of time spent on self‐care activities can have health implications; hence, it is important to find ways to allocate this time appropriately. This reduction in personal care time is concerning from an occupational therapy perspective, as adequate self‐care is important for maintaining caregiver health and preventing burnout. Sim et al. found that Chinese mothers of children with disabilities who engaged in fewer health‐promoting activities reported poorer well‐being. Similarly, Bourke‐Taylor et al. showed that maternal health‐promoting behaviors were associated with lower stress among mothers of children with developmental disabilities. These findings highlight the need for interventions that support mothers in maintaining adequate time for personal ADLs alongside caregiving responsibilities.

In addition, mothers of children with disabilities work 141.71 fewer minutes than mothers of children without disabilities. The fact that mothers of children with disabilities spent less time working than mothers of children without disabilities is likely a result of differences in employment status. Looking at the general characteristics of the sample, 97 mothers were economically active (Table 2). Of these, 64 mothers of children without disabilities were economically active compared to 33 mothers of children with disabilities, suggesting that the difference in employment status may explain why mothers of children with disabilities spend less time working. This finding is consistent with research by Dutra et al., who identified maternal employment status as a key determinant of caregiving time allocation.

On the other hand, mothers of children with disabilities spent 148.67 min more on IADLs than mothers of children without disabilities and 158.85 min more on childcare in the subscale. These results were consistent with previous studies that found that mothers of children with disabilities spent more time parenting than mothers of children without disabilities [31]. Sim et al. also found that mothers of children with disabilities in Australia, Singapore, and Taiwan experienced greater caregiving demands. This finding demonstrates that the pattern of reduced self‐care time affecting maternal health transcends cultural contexts, as mothers across different countries experience similar challenges when caring for children with disabilities. Other studies have also found that mothers of children with disabilities spend a lot of time raising their children, especially in terms of sleep and social and cultural activities [9]. Thus, the long time spent caring for their children means that they are sacrificing time in other areas. For mothers of children with disabilities to have a more balanced use of time, measures to reduce the burden of childcare are needed. Occupational therapy–based time management programs and caregiver education interventions have shown promise in helping mothers achieve better occupational balance [17]. In addition, parents of children with disabilities have psychological and emotional difficulties [33], and as children with disabilities require a lot of help in daily life, the long hours of parenting increase these difficulties; therefore, there is a need for institutional support for parents of children with disabilities, such as through programs to promote psychological and emotional stability.

Mothers of children with disabilities spent 17.33 min more time communicating with the healthcare system than mothers of children without disabilities, which is likely due to more time spent in healthcare institutions, such as hospitals, for consultations and communication about the treatment of their children with disabilities. Mothers may also tend to spend more time in healthcare institutions as caregivers, as previous research has shown that children with disabilities spend more time in healthcare institutions for treatment than in childcare and education institutions [34].

Next, we examined mothers′ time deficiency according to whether their child had a disability or not and observed a significant difference in time deficiency between groups, with mothers of children with disabilities feeling more time deficient (3.09 vs. 2.61, *p* < 0.001). Previous studies have demonstrated that parents of children with disabilities report that they are limited in their activities due to the burden of caring for their children and that they have difficulty managing their time [35, 36]. Time deficiency is a psychological state in which individuals feel that they have less time than they need [22]. Mothers of children with disabilities spend a lot of their time caring for their children, which leaves them with less time for other areas of their lives. Therefore, mothers of children with disabilities seem to feel more time‐deficient. This heightened sense of time deficiency likely reflects not only objective time constraints but also the psychological experience of occupational imbalance. Research has established that chronic time pressure and time scarcity are associated with adverse health outcomes, including increased stress, anxiety, poorer mental health, and reduced physical well‐being [24]. Time deficiency can act as a physical and psychological stressor, leading to sleep difficulties, fatigue, and diminished capacity for self‐care behaviors such as exercise and healthy eating [37, 38]. For mothers of children with disabilities, these health risks may be particularly pronounced given their elevated baseline stress levels and reduced time for health‐promoting activities, where caregiving demands reduce time for other meaningful occupations, including self‐care, leisure, and social participation. Considering that other studies have shown that mothers of children with disabilities have higher time management satisfaction and lower parenting stress when they receive appropriate support [39], comprehensive support strategies are needed for mothers of children with disabilities. These may include respite care services to provide temporary relief from caregiving demands, flexible workplace policies that accommodate caregiving responsibilities, peer support groups to reduce isolation, and access to coordinated care systems that streamline healthcare appointments. While time management skills can be beneficial, the primary need is for structural support that addresses the systemic barriers these mothers face rather than placing the burden solely on individual time management.

When we examined the relationship between mothers′ time use and time deficiency according to whether their child had a disability or not, we found that IADLs had the highest static correlation with time deficiency among mothers of children with disabilities. This means that the more time mothers spend on IADLs, which includes time spent caring for their children, the more time they feel they are running out of. Conversely, time deficiency and ADLs had the highest negative correlation, suggesting that the more time spent on ADLs, the less time deficiency. This suggests that more time for basic daily activities, such as self‐care and eating, may reduce mothers′ feelings of time deficiency and provide them with a sense of psychological well‐being. Therefore, support is essential to help mothers improve their quality of life through balanced work engagement. These findings have practical implications for occupational therapy interventions, suggesting that strategies to protect and increase mothers′ time for personal ADLs may reduce their perceived time deficiency.

In terms of the correlation between mothers of children without disabilities′ time use and time deficiency by occupation area, education had the highest positive correlation. This means that the more time mothers spend on education, the more time they feel they are running out of. Rest and sleep, on the other hand, had the highest negative correlation, indicating that the more time spent resting and sleeping, the less time deficiency mothers felt.

Next, we examined the effect of mothers′ time use in different occupation areas on time deficiency according to whether their child had a disability or not. First, we found that daily activities negatively affected time deficiency for mothers of children with disabilities. This means that the more time mothers spend on daily activities, the less time deficiency they feel. It appears that it is important for mothers of children with disabilities to provide opportunities for them to use and utilize time for themselves in addition to the time they spend raising their children. In addition, IADLs were positively associated with time deficiency, but the effect was relatively insignificant. On the other hand, when the effect of time use by occupation area was examined for mothers of children without disabilities, time use by area did not have a significant effect on time deficiency.

The present study examined the effect of mothers′ general characteristics on time deficiency, distinguishing between groups based on the presence or absence of a disability in their child. The analysis revealed that, for both groups, increasing mothers′ age was associated with a positive correlation between age and time deficiency. This finding aligns with prior studies that have identified a correlation between mothers′ age and perceived time constraints [40].

While this study measured time use objectively through time diaries and time deficiency as a subjective psychological state, it is important to consider how these constructs relate to the broader concept of occupational balance within the OTPF. Time use represents the objective allocation of hours across activities, whereas time deficiency captures the subjective feeling that available time is insufficient. Occupational balance, in contrast, reflects the quality and appropriateness of engagement across diverse occupational domains—a state that supports well‐being even when objective time is constrained.

Our findings suggest that for mothers of children with disabilities, it is not only the amount of time spent on caregiving that matters, but how this time allocation disrupts balance across occupations. The strong negative correlation between ADL time and time deficiency among mothers of children with disabilities suggests that maintaining time for self‐care—even in small amounts—may be psychologically beneficial. This is consistent with the OTPF′s emphasis on balanced occupational engagement as important for health and quality of life. However, applying the OTPF to analyze caregiving patterns was conceptually challenging, as caregiving occupations often blur boundaries between IADL (child rearing) and other domains, and the framework does not fully capture the intensity or emotional aspects of disability caregiving. Future research may benefit from developing more detailed occupational classifications specific to caregiving contexts.

Occupational balance refers to a state in which individuals are able to meaningfully and appropriately engage in a variety of occupations in their daily lives and is closely related to components of well‐being such as emotional stability, self‐satisfaction, and quality of life. In particular, mothers of children with disabilities are prone to experiencing occupational imbalance due to the time demands of caregiving, which, along with perceived time deficiency, may contribute to reduced well‐being. In light of this, the findings of this study can serve as a foundational evidence base for designing targeted interventions and social policies that are aimed at alleviating time deficiency among mothers of children with disabilities. Strategies such as the implementation of caregiving support services, occupational balance–based educational programs, and community‐based respite care systems may enhance these mothers′ well‐being by promoting more equitable time use.

Some limitations of this study include the geographical area of the subjects and the type of child′s disability; the length of the survey period, which may have resulted in differences in the subjects′ time use at different times of the survey; the small number of subjects, which made it difficult to generalize the results of the study; the fact that the study only examined time use on weekdays without dividing the time between weekdays and weekends; the cross‐sectional nature of the study, which makes it difficult to discern changes in participants′ perceptions of time scarcity over time; and the possibility of selection bias because the participants are required to complete the questionnaire themselves. However, despite these limitations, we were able to find out the differences in mothers′ work and time deficiency according to the presence or absence of their children′s disabilities by dividing the occupation areas based on the OTPF fourth edition and finding out how time use by occupation area and general characteristics of the subjects affect mothers of children with disabilities′ time deficiency. Accordingly, it was found that mothers of children with disabilities felt a greater time deficiency, and it is significant that this can be used as a basis for establishing a plan to resolve the time deficiency of mothers of children with disabilities by appropriately distributing weighted or insufficient occupation.

## 5. Conclusions

When mothers′ time use was examined by whether their child had a disability or not, mothers of children with disabilities spent more time raising their children and communicating with the healthcare system, while mothers of children without disabilities spent more time eating, attending to personal hygiene and grooming, and performing and maintaining work.

A comparison of time deficiency between the two groups showed that mothers of children with disabilities felt more time‐deficient, and the more time they spent on daily activities had a decreasing effect on time deficiency. Contrarily, for mothers of children without disabilities, the amount of time spent in each occupation area did not affect time deficiency.

Based on these findings, mothers of children with disabilities felt more time deficiency and had difficulty balancing their occupation. These findings suggest that mothers of children with disabilities may need extensive support to enable them to use and utilize their time for themselves, in addition to the time they spend raising their children. Accordingly, further research is recommended to build on the current study′s findings and develop more ways to address mothers′ time use challenges. It is our hope that subsequent research will build upon the findings of the present study. Such subsequent research might include a longitudinal study that explores the impact of time use in occupation areas on mothers′ feelings of time deficiency over the long term, as opposed to a cross‐sectional design. Additionally, a time use intervention study for mothers of children with disabilities who are experiencing feelings of time deficiency might be a fruitful avenue for future research. Such interventions could include respite care programs, coordinated care models that reduce appointment burden, workplace flexibility initiatives, peer support networks, and occupational therapy–based approaches that help mothers identify and prioritize meaningful occupations beyond caregiving. Future research should also explore the effectiveness of policy interventions such as paid family leave, subsidized childcare for children with special needs, and integrated service delivery systems that minimize time spent navigating fragmented healthcare and educational systems.

## Funding

No funding was received for this manuscript.

## Conflicts of Interest

The authors declare no conflicts of interest.

## Data Availability

The data that support the findings of this study are available on request from the corresponding author. The data are not publicly available due to privacy or ethical restrictions.
